# *Brachybacterium conglomeratum* Is Associated with Cervicovaginal Infections and Human Papilloma Virus in Cervical Disease of Mexican Female Patients

**DOI:** 10.3390/microorganisms11112769

**Published:** 2023-11-14

**Authors:** Iliana Alejandra Cortés-Ortíz, Jonathan Puente-Rivera, Guiedaana Ordaz-Pérez, Alejandra Yareth Bonilla-Cortés, Paula Figueroa-Arredondo, Carlos Alberto Serrano-Bello, Eduardo García-Moncada, Gustavo Acosta-Altamirano, Daniel Ernesto Artigas-Pérez, Juan Carlos Bravata-Alcántara, Mónica Sierra-Martínez

**Affiliations:** 1 Genetic and Molecular Diagnostic Laboratory, Hospital Juárez de México, Instituto Politécnico Nacional 5160, Col. Magdalena de las Salinas, Mexico City 07360, Mexico; iliancortes@yahoo.com.mx (I.A.C.-O.); ordaz.daana@gmail.com (G.O.-P.); eduardo.garcia.moncada@gmail.com (E.G.-M.);; 2 División de Investigación, Hospital Juárez de México, Instituto Politécnico Nacional 5160, Col. Magdalena de las Salinas, Mexico City 07360, Mexico; jo_puenter@hotmail.com; 3 Escuela Nacional de Medicina y Homeopatia, Instituto Politécnico Nacional, Av. Guillermo Massieu Helguera 239, La Purísima Ticomán, Gustavo A. Madero, Mexico City 07320, Mexico; yarethcortes1998@gmail.com; 4 Escuela Superior de Medicina, Instituto Politecnico Nacional, Salvador Diaz Mirón esq. Plan de San Luis S/N, Miguel Hidalgo, Casco de Santo Tomas, Mexico City 11340, Mexico; paula_figueroa@outlook.com; 5 Servicio de Unidad Patológica, Hospital Juárez de México, Instituto Politécnico Nacional 5160, Col. Magdalena de las Salinas, Mexico City 07360, Mexico; crls.serrbe@gmail.com; 6 Unidad de Investigación en Salud, Hospital de Alta Especialidad Ixtapaluca, Carr Federal México-Puebla Km 34.5, Ixtapaluca 56530, Mexico; mq9903@live.com.mx

**Keywords:** microbiota, *Brachybacterium conglomeratum*, HPV, low-grade squamous intraepithelial lesion

## Abstract

*Brachybacterium conglomeratum*, traditionally considered an environmental bacterium, has recently garnered attention for its potential involvement in human health. While prior research hinted at its pathogenic role in humans, our study aims to determine its prevalence and associations in diverse clinical contexts. We examined vaginal swabs from three distinct patient groups: patients with low-grade squamous intraepithelial lesions (LSIL), patients with cervicovaginal infections, and patients with a history of precancerous lesions undergoing follow-up. *B. conglomeratum* was present in all three patient groups, with the highest prevalence observed in the LSIL group. Statistically significant associations were primarily identified in the LSIL group, where *B. conglomeratum* was present in 60% of cases. Notably, the LSIL group exhibited coinfections with multiple high-risk oncogenotypes of human papillomavirus (HPV), suggesting potential synergistic effects, and understanding these microbial relationships and their influence on viral persistence, particularly with HPV, holds promise for mitigating HPV-related carcinogenesis. Furthermore, *Gardnerella vaginalis* and *Atopobium vaginae* were frequently detected in this group, along with *Ureaplasma parvum* as the predominant sexually transmitted bacterium. In all cases, *B. conglomeratum* was found in association with these microorganisms rather than as a sole pathogen. This coexistence underscores the intricate microbial interactions within cervicovaginal infections and precancerous lesions. This study marks the first report of *B. conglomeratum* prevalence in women with these clinical conditions.

## 1. Introduction

The normal human vagina is a balanced microenvironment in which the microbiota plays a crucial regulatory role. *Lactobacillus* spp. are the bacteria primarily responsible for preventing colonization by other pathogenic microorganisms that could disrupt this balance. When this microbiota is present in low concentrations or absent, the microbial diversity, especially of anaerobes, increases, making the epithelial tissue more susceptible to colonization by external microorganisms [1,2,3].

Consequently, infections with sexually transmitted bacteria and bacterial vaginosis can find an excellent niche for implantation. *Gardnerella vaginalis* and *Atopobium vaginae* have been considered markers of abnormal vaginal microbiota [4], as they actively contribute to the generation of an unstable microenvironment [5,6]. Moreover, increased microbial diversity is also associated with viral persistence and progression to cervical cancer in oncogenic human papillomavirus (HPV) infections [7].

More than 200 HPV genotypes have been identified, each displaying distinct preferences and specificities in regard to invading the host [8]. For instance, among the primary HPV genotypes responsible for cervical cancer, HPV18 and HPV52 are prominent, while HPV16 is the primary HPV genotype associated with squamous cell carcinoma [9]. A total of 13 HPV genotypes are definitively linked to oncogenic risk and have been categorized as high risk by the International Agency for Research on Cancer. HPV 16 and 18 come with an elevated risk and therefore should be managed differently from the other 11 genotypes. Genotypes 31, 33, 52, and 58 confer risks similar to HPV 18 and 45, which provide a basis for considering more complex screening algorithms involving genotype-specific risk stratification [10]. Consequently, HPV genotypes 16, 18, and 45 warrant closer surveillance than women infected with other high-risk HPV genotypes. However, there is a growing trend in risk stratification through genotyping to enhance the triage of existing cytology-based screening, and proposals have been put forward to address women with ASC-US or low-grade squamous intraepithelial (LSIL) cytology. Several studies have demonstrated that HPV infection is linked to an imbalance in vaginal microbial composition. Furthermore, HPV infection has been shown to alter the richness and diversity of the vaginal microbiota [11].

Studies have reported a direct relationship between the vaginal microbiome and racial groups [12]. Specific bacterial groups have been found in Caucasian, Afro-descendant [13], and Japanese [14] women and some of these bacterial groups may be related to severe pathologies [15]. Therefore, depending on the biome, susceptibility to certain sexually transmitted diseases may increase or decrease [16].

In a study of the vaginal microbiome in Mexican women with low- and high-grade cervical intraepithelial lesions, an association of *Brachybacterium conglomeratum* with HPV was reported [17]. This finding suggests that *B. conglomeratum* could serve as a potential biological marker for detecting precancerous cervical lesions in this population. This bacterium had been considered an environmental microorganism [18] until it was detected in a case of bacteraemia [19] and in a postcataract endophthalmitis case [20]. However, there are no reports elucidating the pathogenic role of *B. conglomeratum* and its relationship with pathogenic microorganisms in the female genital tract.

The objective of this study was to determine the frequency of *B. conglomeratum* in vaginal swabs of Mexican patients with dysbiosis and to describe its interrelation with sexually transmitted bacteria and bacterial vaginosis. Additionally, the study aimed to confirm the association of this bacterium with HPV in patients with low-grade squamous intraepithelial lesions (LSILs).

## 2. Materials and Methods

### 2.1. Sample Collection

Cervical exudate samples (194) were collected from Mexican patients who attended the colposcopy service between January 2022 and May 2022. The present study was approved by the Ethics and Research Committee of the Hospital Regional de Alta Especialidad Ixtapaluca (NR-014-2022), and the samples were collected using BD Universal viral transport medium for viruses, *Chlamydiae, Mycoplasma*, and Ureaplasmas (Becton, Dickinson and Company, Sparks, MD, USA). Written informed consent was obtained from all patients.

### 2.2. Grouping of Patients According to Type of Cervicovaginal Lesions

From a total of 194 patient samples analysed, 99 exudates were obtained from women with cervicovaginal infections and no epithelial lesions (nonlesion, Group 1), 42 exudates were from patients with low-grade squamous intraepithelial lesions (LSIL) confirmed by histology (LSIL, Group 2), and 53 exudates were from patients who underwent cone biopsy or surgery due to LSIL and only attended for cervical cancer control (CCC, Group 3). The age range of 25–29 years had the highest number of cases.

### 2.3. Molecular Detection and Characterization of HPV and Sexually Transmitted Pathogens

Total DNA from patients with vaginal swabs was isolated using the Invisorb Spin Universal Kit (Invitek Molecular GmbH, Berlin, Germany) following the manufacturer’s instructions. One microgram of DNA was used for qPCR amplification. Two commercial kits were employed for the identification of HPV and bacterial sexually transmitted infections: HPV28 Detection (AnyplexTM II, HP7S00X, MT Promedt Consulting GmbH, St. Ingbert, Germany) and STI-7 Detection (v1.1) (AnyplexTM II, SD7700Y, MT Promedt Consulting GmbH, St. Ingbert, Germany). The HPV28 kit detects 19 high-risk human papillomavirus genotypes, such as 16 (HPV 16), 18 (HPV 18), 26 (HPV 26), 31 (HPV 31), 33 (HPV 33), 35 (HPV 35), 39 (HPV 39), 45 (HPV 45), 51 (HPV 51), 52 (HPV 52), 53 (HPV 53), 56 (HPV 56), 58 (HPV 58), 59 (HPV 59), 66 (HPV 66), 68 (HPV 68), 69 (HPV 69), 73 (HPV 73), 82 (HPV 82), and 9 low-risk human papillomavirus genotypes, such as 11 (HPV 11), 40 (HPV 40), 42 (HPV 42), 43 (HPV 43), 44 (HPV 44), 54 (HPV 54), 6 (HPV 6), 61 (HPV 61), 70 (HPV 70), and their respective internal control (IC). The STI-7 kit detects *Neisseria gonorrhoeae* (N.g.), *Chlamydia trachomatis* (Ch.t.), *Mycoplasma genitalium* (M.g.), *Mycoplasma hominis* (M.h.), *Ureaplasma urealyticum* (U.u.), *Ureaplasma parvum* (U.p.), and *Trichomonas vaginalis* (T.v.). The procedures were carried out according to the manufacturer’s instructions using the following PCR cycling conditions: initial polymerase activation step (95 °C, 10 min), 35 cycles at 95 °C, 30 s, 55 °C, 15 s, and 72 °C for 30 s.

For the identification of *B. conglomeratum*, *Gardnerella vaginalis*, and *Atopobium vaginae*, specific primers and probes were designed for qPCR identification as indicated in Table 1.

The primer sequences and probe were obtained using Primer3Plus software (https://www.bioinformatics.nl/cgi-bin/primer3plus/primer3plus.cgi, accessed on 25 January 2022), the specificity of the primers and probes was evaluated in silico, and amplification was performed using PCR master mix (Luna, Universal Probe PCR Master Mix, M3004L, New England BioLabs Inc., Santa Barbara, CA, USA), with 10 pM of each forward and reverse primer, 1 pM of the probe, and 1 µg of each DNA sample as the template. PCR was performed using a CFX96 Real-Time System (Bio-Rad, Hercules, CA, USA) under the following conditions: denaturation at 95 °C for 3 min, denaturation at 95 °C for 10 s, and annealing and extension at 58 °C for 30 s for 39 cycles.

### 2.4. Statistical Analysis

To establish whether *B. conglomeratum* is independent or related to sexually transmitted bacteria and/or HPV in patients with dysbiosis, the chi-square test (χ^2^) was employed to assess the correlation of variables, considering a *p* value of <0.05 as statistically significant. To find the type of relationship (strong, moderate, or weak) and the direction of the relationship, Kendall’s Tau test and bivariate normal correlation were used in IBM SPSS software (version 28.0.1), a commercial platform for statistical analysis. To calculate the statistical power and effect size, the G Power platform (freely available software, version 2009) was used.

## 3. Results

### 3.1. B. conglomeratum Is Detected in Conjunction with HPV and Sexually Transmitted Microbes in Patients with Cervicovaginal Coinfections without LSIL

qPCR testing for the identification of *B. conglomeratum*, HPV, and sexually transmitted bacteria (with a special focus on *G. vaginalis* and *A. vaginae*) was performed on all exudates obtained from these patients with cervicovaginal coinfection but not LSIL. *B. conglomeratum* was found in 58% of the analysed exudates (58/99). HPV detection showed 30% positive cases (30/99), with 19/99 (19%) presenting oncogenic HPV types (39, 45, 53, 56, 58, 59, 73, 82, 66–39, 51–69) and 11/99 (11%) presenting low-risk HPV (6, 42, 43, 44, 54, 61, 70). For sexually transmitted bacteria, 64/99 (65%) were positive, with *U. parvum* being the most frequent (49%), followed by *U. urealyticum* (14%). *T. vaginalis* (1%) was also detected, along with coinfections of *U. parvum*-*U. urealyticum* (3%), *U. parvum*-*M. genitalium* (1%), and *U. urealyticum*-*Mycoplasma hominis* (1%). *G. vaginalis* and *A. vaginae* were detected in 43% and 47% of patients, respectively.

To obtain the interrelation of *B. conglomeratum* with HPV and sexually transmitted pathogens in each analysed group, a Venn diagram was constructed. This diagram illustrates the logical relationships between sets based on presence/absence data and the shared elements between subsets in the analysed groups.

**Figure 1:**
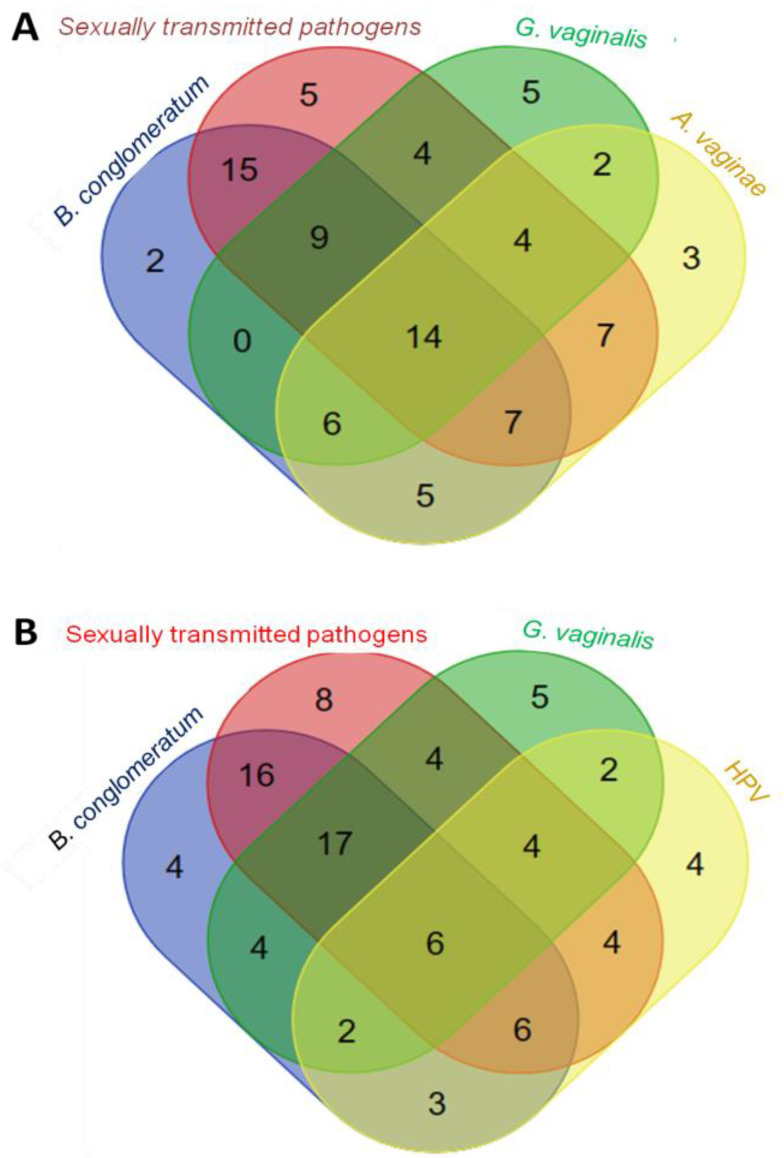
Correlation of *B. conglomeratum* with various cervicovaginal infections using a Venn diagram. (**A**) Overlap of set elements with the sexually transmitted pathogens *G. vaginalis* and *A. vaginae*, (**B**) with *G. vaginalis* and HPV. The numbers in the circles indicate the quantity of elements in the subsets based on presence/absence data. The numbers in the overlapping regions indicate the quantity of elements shared between subsets.

In Group 1, *B. conglomeratum* was mainly associated with two sets. The first association was with sexually transmitted pathogens (set 1), and the second corresponded to the intersection of four elements (*B. conglomeratum*, sexually transmitted pathogens, *G. vaginalis*, and *A. vaginae* (set 2)). Within the association of *B. conglomeratum* with sexually transmitted pathogens, *U. parvum* (10 out of 15 elements, 67%) was the most frequent, followed by *U. urealyticum* (4 out of 15, 27%) and *T. vaginalis* (1 out of 15, 6%). In the association of *B. conglomeratum* with *G. vaginalis*, *A. vaginae*, and the sexually transmitted pathogens *U. parvum* (11 out of 14, 79%), *U. urealyticum* (7%), and *U. urealyticum*/*M. hominis* (7%) were found (Figure 1A).

When the set *A. vaginae* was replaced with the HPV set, the association of elements changed noticeably. It was now observed that the strongest association of *B. conglomeratum* was in the intersection of the set of sexually transmitted pathogens and *G. vaginalis* (with 17 elements), followed by the association of *B. conglomeratum* and sexually transmitted pathogens (16 elements). It was evident that *B. conglomeratum* had a greater association with sexually transmitted pathogens and *G. vaginalis*, and among these, *U. parvum* was the bacterium with the highest detected association (Figure 1B). When relating the set of *B. conglomeratum* and HPV, 26 elements intersected, leaving four elements of *B. conglomeratum* unassociated.

### 3.2. B. conglomeratum Is More Prevalent in Patients with LSIL and HPV Infection

In Group 2 (LSIL patients), *B. conglomeratum* was found in 60% (29/42) of the analysed exudates, and HPV was found in 74% (31/42), with genotypes 16 and 53 being the most frequent, and a 60% (25/42) prevalence of sexually transmitted pathogens were observed. *B. conglomeratum* was detected in 81% (25/31) of HPV, 84% (21/25) of sexually transmitted pathogens, and 40% (17/42) of *G. vaginalis*-positive and 38% (16/42) of *A. vaginae*-positive exudates. In this group, multiple coinfections with oncogenic HPV types, with two to seven HPV genotypes present in some cases (with double-coinfection: 16–18, 16–52, 16–58, 51–53, 59–53, 42–53, 43–61, 61–6, 66–61, 66–56, 70–40; triple-coinfection: 19–59-52, 51–61–53, 31–42–61, 45–11–70, 52–56–53, 66–69–52; and more-coinfection: 58–51–39–52–54–70–44, 59–35–53–42, 31–40–66–53–56–6, 53–45–73–70), and sexually transmitted pathogens were observed. *U. parvum* 24% (4/17), *U. urealyticum* 12% (2/17), *M. hominis* 24% (4/17), and *T. vaginalis* 6% (1/17), and coinfections among sexually transmitted pathogens were detected and included *U. parvum-U. urealyticum* 6% (1/17), *U. urealyticum-M. hominis* 18% (3/17), *U. parvum*-*M. genitalium* 6% (1/17), and *U. urealyticum-C. trachomatis-M. hominis* 6% (1/17). *G. vaginalis* and *A. vaginae* were detected in 76% (13/17) and 88% (14/16) of patients, respectively, in this group.

**Figure 2:**
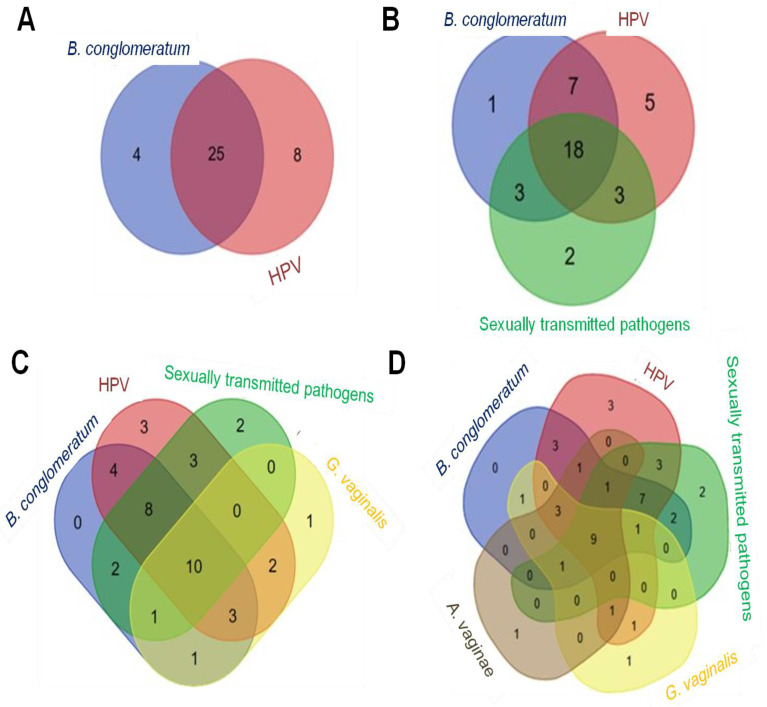
Correlation of *B. conglomeratum* in patients with LSIL. (**A**) Overlap of elements in sets with oncogenic HPV. (**B**) *B. conglomeratum* with sexually transmitted pathogens and HPV. (**C**) *B. conglomeratum* with sexually transmitted pathogens, HPV, and *G. vaginalis*. (**D**) *B. conglomeratum* with sexually transmitted pathogens, HPV, *G. vaginalis*, and *A. vaginae*. The numbers in the circles indicate the quantity of elements in the subsets based on presence/absence data. The numbers in the overlapping regions indicate the quantity of elements shared between subsets. The Venn diagram was created using the Bioinformatics & Evolutionary Genomics webpage URL: http://bioinformatics.psb.ugent.be/webtools/venn/, accessed on 20 January 2023.

When including sexually transmitted pathogens, 18 elements were associated with all three sets, and the 4 unassociated elements from the *B. conglomeratum* set were grouped within the sexually transmitted pathogens set (Figure 2). Regarding Group 2 (LSIL), *B. conglomeratum* was associated with oncogenic HPV types in 25/32 swabs (78%), including all detected oncogenotypes of HPV 16 (Figure 2A). When including the set of sexually transmitted pathogens in the relationship between *B. conglomeratum* and HPV, it was observed that out of the 25 samples from the intersection of *B. conglomeratum*-HPV, 18/25 (72%) were associated, with *M. hominis* being the most common (44%), followed by *U. urealyticum* at 39%, *U. parvum* at 33%, *C. trachomatis*, *M. genitalium*, and *T. vaginalis* at 6% each. Multiple coinfections with different high-risk HPV genotypes (33%) and sexually transmitted pathogens (22%) were also detected in this group (Figure 2B). When including *G. vaginalis* and *A. vaginae* in the set of *B. conglomeratum*-HPV-sexually transmitted pathogens, the 7 elements at the intersection of *B. conglomeratum* with HPV were rearranged because three of them were associated with *G. vaginalis* and *A. vaginae*, and only one was associated with *A. vaginae* (Figure 2D). The elements of *B. conglomeratum* that were not associated with any previous set were now linked to *G. vaginalis*, indicating that *B. conglomeratum* is consistently found in coinfection with other pathogens. Out of the 18 elements found at the intersection of *B. conglomeratum*, HPV, and sexually transmitted pathogens, 10 intersected with *G. vaginalis* (Figure 2C). Adding *A. vaginae*, nine elements were associated with all sets (Figure 2D).

### 3.3. B. conglomeratum Is Also Detected in Cervical Cancer Control Patients

In the group of patients who underwent cone biopsy or surgery for cervical cancer control (CCC, Group 3), *B. conglomeratum* was found in 36% of the analysed exudates (19/53). Twenty of 53 (23%) had HPV, with 6/53 (11%) being high-risk HPV, 2/53 (4%) having double HPV coinfections (58–68 and 56–68), and 4/53 having low-risk HPV. Sexually transmitted bacteria were detected in 23% of these exudates, with *U. parvum* being present in 23% and *U. urealyticum* in 2%. No other pathogen coinfections were found in this group.

**Figure 3:**
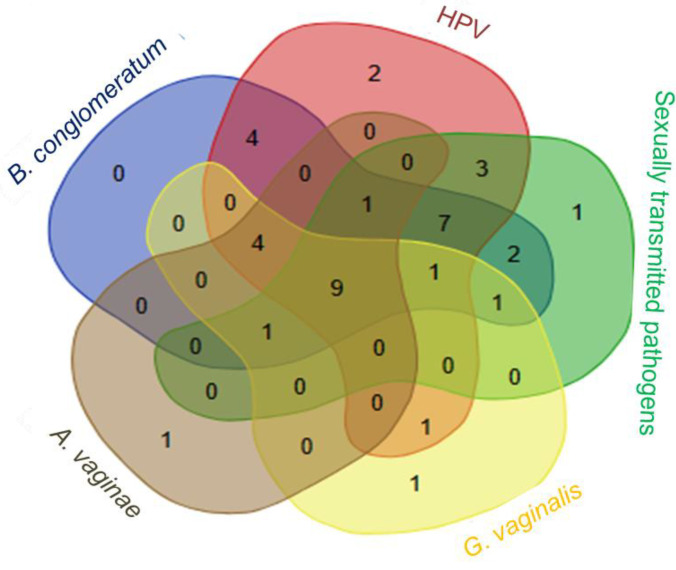
Correlations of *B. conglomeratum* in patients from the posttreatment group, illustrating the overlap of elements in sets with sexually transmitted pathogens, HPV, *G. vaginalis*, and *A. vaginae*. The numbers in the circles indicate the quantity of elements in the subsets based on presence/absence data. The numbers in the overlapping regions indicate the quantity of elements shared between subsets. The Venn diagram was created using the Bioinformatics & Evolutionary Genomics webpage URL: http://bioinformatics.psb.ugent.be/webtools/Venn/, accessed on 20 January 2023.

Out of the 53 vaginal swabs from patients who underwent cone biopsy or surgery for cervical cancer control (CCC, Group 3), *B. conglomeratum* was found in 20 samples (37%). Of these, 6 were associated with HPV. *B. conglomeratum* was related to 3 elements with sexually transmitted pathogens, six elements with *G. vaginalis*, and seven elements with *A. vaginae*. Only one sample intersected with all the studied sets (Figure 3).

### 3.4. B. conglomeratum Is Associated with Sexually Transmitted Pathogens and HPV

To determine whether *B. conglomeratum* is independent or has a statistically significant relationship in the evaluated Groups 1, 2, and 3, a chi-square test was conducted. For the group of sexually transmitted bacteria and *B. conglomeratum*, a *p* value of 0.002 was obtained, indicating a statistical relationship. However, no statistical relationship was found with HPV (Table 2, Group 1).

In the LSIL group, the association of *B. conglomeratum* with oncogenic HPV types and sexually transmitted bacteria also showed a statistical relationship with these bacteria and HPV (Table 2, Group 2). However, in the posttreatment group, this statistically significant relationship between *B. conglomeratum*, HPV, and sexually transmitted bacteria was not found, suggesting that this bacterium is not related to these groups (Table 2, Group 3).

To search for a possible correlation between two variables, the Kendall’s Tau-b test was used, where it was found that the association between *B. conglomeratum* and Group 1 had a value of 0.306, corresponding to a medium-low statistical association (Table 3, Group 1). For Group 2, the association between *B. conglomeratum* and HPV was also medium-low (correlation of 0.0316), but in contrast, this group showed a medium-high association between *B. conglomeratum* and sexually transmitted pathogens (correlation of 0.446) (Table 3, Group 2). No significant association was found between STP and HPV in Group 3 (Table 3, Group 3).

To assess the impact of these associations, a bivariate normal model was used with the previous values to calculate the effect size, which indicates the magnitude of the effect under an alternative hypothesis. Using Cohen’s rules for effect size classification, where a small effect is 0.2, medium is 0.5, and large is 0.8, the following was determined: 1. For the association between *B. conglomeratum* and sexually transmitted pathogens in Group 1, the effect size was 0.5531, which is considered medium. 2. No significant association was found between *B. conglomeratum* and HPV in Group 1. 3. In Group 2, the correlation between *B. conglomeratum* and sexually transmitted pathogens had an effect size of 0.6678, indicating a medium-high effect. The correlation between *B. conglomeratum* and HPV in Group 2 had an effect size of 0.5621, indicating a medium effect. 4. Only the association between *B. conglomeratum* and sexually transmitted pathogens in Group 2 represented a medium-high effect.

Using statistical power (1-β error prob), in Group 1, the alternative hypothesis was accepted for the lesion-free group, as there was a correlation between B. conglomeratum and sexually transmitted pathogens, with a β value of 0.0014 and a power of 0.9986. No significant correlation was found between *B. conglomeratum* and HPV in Group 1. In Group 2, the correlation between *B. conglomeratum* and sexually transmitted pathogens resulted in β = 0.0184 and a power of 0.9816, and the correlation between *B. conglomeratum* and HPV had β = 0.0169 and a power of 0.9831. No statistical power was found in Group 3 for the correlation between *B. conglomeratum* and HPV. All correlations were adequate, indicating that the alternative hypothesis suggests that the chi-square correlations and Kendall’s Tau-b correlations are representative of these analysed groups (Table 4).

Using the correlation data *for B. conglomeratum* and infection, a two-tailed bivariate normal distribution diagram was constructed using the G Power program, resulting in a 1-β error probabilistic value of 0.99. This value is associated with statistical power when making population inferences. With this obtained value, the correlation was generalized by calculating the effect using the normal distribution, resulting in a value of 0.394, indicating a medium effect according to the value scale, which can be considered clinically relevant.

## 4. Discussion

*Brachybacterium conglomeratum* has been considered an environmental bacterium, and few studies have demonstrated its pathogenic role in humans [19,20]. However, in 2021, it was detected in Mexican patients infected with HPV 16 and with low-grade squamous intraepithelial lesions (LSIL), suggesting it as a possible biological marker for LSIL [17]. Thus, in the present study, we examined the prevalence of *B. conglomeratum* not only in vaginal swabs from patients with LSIL but also in patients with cervicovaginal infections and patients who had previously experienced precancerous lesions and were currently attending the hospital for follow-up. This is the first study to report the prevalence of this bacterium in women with these two clinical conditions.

*B. conglomeratum* was found in all three groups of patients studied, with a higher prevalence in the group of women with LSIL, followed by the group of patients with cervicovaginal infections. However, statistically, *B. conglomeratum* was found only to be associated only with the group of patients with LSIL. In this group, *B. conglomeratum* was found in 60% of cases, and 16 high-risk HPV oncogenotypes were detected, with HPV 16 and 53 being the most common genotypes, followed by genotypes 51, 52, and 59. All women with HPV genotype 16 were associated with *B. conglomeratum*, confirming the previously reported result by Nieves-Ramirez et al., 2021. This group had the highest number of multiple infections with different high-risk HPV oncotypes compared to the other groups evaluated, which is consistent with the findings of González-Yebra et al. in 2022, who reported that the highest number of HPV genotypes is found in patients with precancerous lesions, and these tend to decrease in high-grade lesions [21].

The fact that we found multiple coinfections with genotypes 16 and 53 in our results suggests the synergistic ability of these genotypes to coexist with other HPV types. Del Prete et al., 2019, reported this coexistence with other HPV genotypes in Italian women [22].

*G. vaginalis*, along with *A. vaginae*, were the bacteria most frequently found in this group, and *U. parvum* was the most common sexually transmitted pathogen, followed by *M. hominis*. Within these groups, *B. conglomeratum* was always found in association with these microorganisms and was not detected as a sole pathogen. It has been observed that *U. parvum, U. urealyticum*, and *M. hominis* increase the risk of abnormal cervical cytology, while *U. urealyticum* and *M. genitalium* increase HPV colonization by influencing the development of cytological abnormalities caused by this virus [23,24]. It is important to reconsider the bacteria that may or may not be associated with pathological conditions, as is the case with *A. vaginae* and *G. vaginalis*. These bacteria have been reported to be of significance in the uterine cervix and can be considered a normal part of the cervical microbiome. HPV infection alone could trigger a significant change in the abundance of bacterial populations. While the mere presence of these bacteria may not be associated with pathological conditions such as the presence of precursor lesions and HPV infection, there is a possibility that an increase in the bacterial load of these genera could play a role in some cervicovaginal diseases [25,26].

The group with the lowest prevalence of *B. conglomeratum* was the one in which patients attended the hospital for follow-up, and 23% were also positive for HPV. Oyervides-Munoz et al., 2020, reported the persistence or recurrence of HPV infection in some patients after one year of treatment due to untreated subclinical HPV infections [27], as well as coinfections with other pathogens such as *Atopobium* and *G. vaginalis* [28]. They validated the molecular PCR test to monitor therapeutic outcomes for these types of infections [29], as cervical vaginal cytology is a highly specific but poorly sensitive technique for detecting recurrences [30]. The vaginal microbial niche is a constantly changing ecosystem comprising over 200 bacterial species that are influenced by genetic factors, ethnic background, and environmental behaviours. Multiple lines of evidence have extensively documented the continuous fluctuations in the vaginal microbiome throughout a woman’s life [31], and the main advantage of probiotic lactobacilli is their ability to restore a healthy, natural microbiome in the vagina, transitioning it from a disease-promoting, imbalanced ecosystem to a healthy, mutually beneficial microbiome [32]. 16S rRNA gene and metagenomic sequencing reveal variations in microbial composition and function within the cervical microbiota between HPV-infected and uninfected women. Metagenomic sequencing can serve as a viable alternative to compensate for the limitations of 16S rRNA gene sequencing technology or to provide mutual validation. The composition and function of the cervical microbiome in HPV-infected individuals markedly differed from that of uninfected women. Uncommon bacteria, such as *Lactobacillus crispatus*, *L. jensenii,* and *L. helveticus*, were identified as predominant species and could serve as biomarkers distinguishing between different groups. These findings may also identify potential microbial targets for future treatment [33]. This approach could provide an alternative means of exploring the association between sexually transmitted pathogens identified in this study and HPV genotypes, especially those associated with LSIL in the Mexican population.

## 5. Conclusions

In our study, *B. conglomeratum* appeared to have no pathogenic capacity per se, as it was always found in coinfection with other sexually transmitted pathogens with a role in cervicovaginal infections or precancerous lesions. However, it could be considered an important coadjutant within these dysbiotic processes, as the interrelation of bacterial groups can create suitable niches for viral persistence. Understanding these relationships and eliminating them could contribute to reducing HPV-related carcinogenesis.

## Figures and Tables

**Table 1:** Primer sequences and gene targets used to detect *B. conglomeratum*, *G. vaginalis* and *A. vaginae*.

Organism	Sequence Target	Primer Sequence
*B. conglomeratum*	16S ribosomal RNA gene (GenBank ID: MT757994, MT760046, NR_104689.1, MN061004.1, MT760046.1)	F:5′- GAAATGCGCAGATATCAGGAAGA-3′
		R: 5′-ACGTTTACGGCATGGACTAC-3′
		P: AGAAGCGAAAGCATGGGTAGCGAA-FAM
*G. vaginalis*	Heat shock protein 60 (hsp60) gene sequence (NCBI GenBank Access: AF240579.3)	F: 5′-AATCTCTGGTGCACGAAGGC-3′
		R: 5′-ACATCCTTAGCAGATGCGAGA-3′
		P: AGCAACCCGATCGCTCTTCGTCGCGGA-FAM
*A. vaginae*	16S ribosomal RNA sequence of strain VCE255 (NCBI GenBank Access number: MH628052.1)	F: 5′-CCTTACCAGGGCTTGACATTTA-3′
		R: 5′-CGGGACTTAACCCAACATCTC-3′
		P: AAGGAGCCTAAACAGGTGGTGCAT-FAM

F: Forward primer. R: reverse primer, P: probe.

**Table 2:** Bilateral statistical relationship of the evaluated groups with *B. conglomeratum*.

Group	Variables	Value	Statistical Significance
Nonlesion (1)	*B. conglomeratum*/STP	9.254	0.002
	*B. conglomeratum*/HPV	0.317	0.573
LSIL (2)	*B. conglomeratum*/STP	8.351	0.004
	*B. conglomeratum*/HPV	4.22	5.67
CCC (3)	*B. conglomeratum*/STP	2.482	0.115
	*B. conglomeratum*/HPV	0.051	0.821

LSIL: low-grade squamous intraepithelial lesion group; STP: sexually transmitted pathogens; HPV: human papilloma virus; CCC: cervical cancer control group.

**Table 3:** Correlation between two variables in the evaluated groups with *B. conglomeratum*.

Group	Variables	Value	Bilateral Statistical Significance
Non-LSIL (1)	*B. conglomeratum*/STP	9.254	0.002
	*B. conglomeratum*/HPV	0.317	0.573
LSIL (2)	*B. conglomeratum*/STP	8.351	0.004
	*B. conglomeratum*/HPV	4.22	5.67
CCC (3)	*B. conglomeratum*/STP	2.482	0.115
	*B. conglomeratum*/HPV	0.051	0.821

LSIL: low-grade squamous intraepithelial lesion group; STP: sexually transmitted pathogens; HPV: human papilloma virus; CCC: cervical cancer control group.

**Table 4:** Bivariate correlation (effect size and power) statistics of the evaluated groups with *B. conglomeratum*.

Group	Variables	Effect Size	Statistical Power
Nonlesion (1)	*B. conglomeratum*/STP	0.5531	0.9986
	*B. conglomeratum*/HPV	NA	NA
LSIL (2)	*B. conglomeratum*/STP	0.6678	0.9816
	*B. conglomeratum*/HPV	0.5621	0.9831
CCC (3)	*B. conglomeratum*/STP	NA	NA
	*B. conglomeratum*/HPV	NA	NA

LSIL: low-grade squamous intraepithelial lesion group; STP: sexually transmitted pathogens; HPV: human papilloma virus; CCC: cervical cancer control group. NA: not associated.

## Data Availability

Publicly available datasets were analysed in this study. These data can be found here: https://www.ncbi.nlm.nih.gov/data-hub/taxonomy/2697049 (accessed on 13 December 2021).
